# Supplementary material for: The interactome of intact mitochondria by cross-linking mass spectrometry provides evidence for coexisting respiratory supercomplexes
Source: Mol Cell Proteomics. 2017 Dec 8;17(2):216–32. doi: 10.1074/mcp.RA117.000470 (PMC5795388; doi:10.1074/mcp.RA117.000470)
Supplement: Supplemental Data [file supp_RA117.000470_133922_0_supp_23978_fzffwf.zip › spectra_annotation/mito_DR_spectra_annotation/76-1-8-1-8-1.pdf]

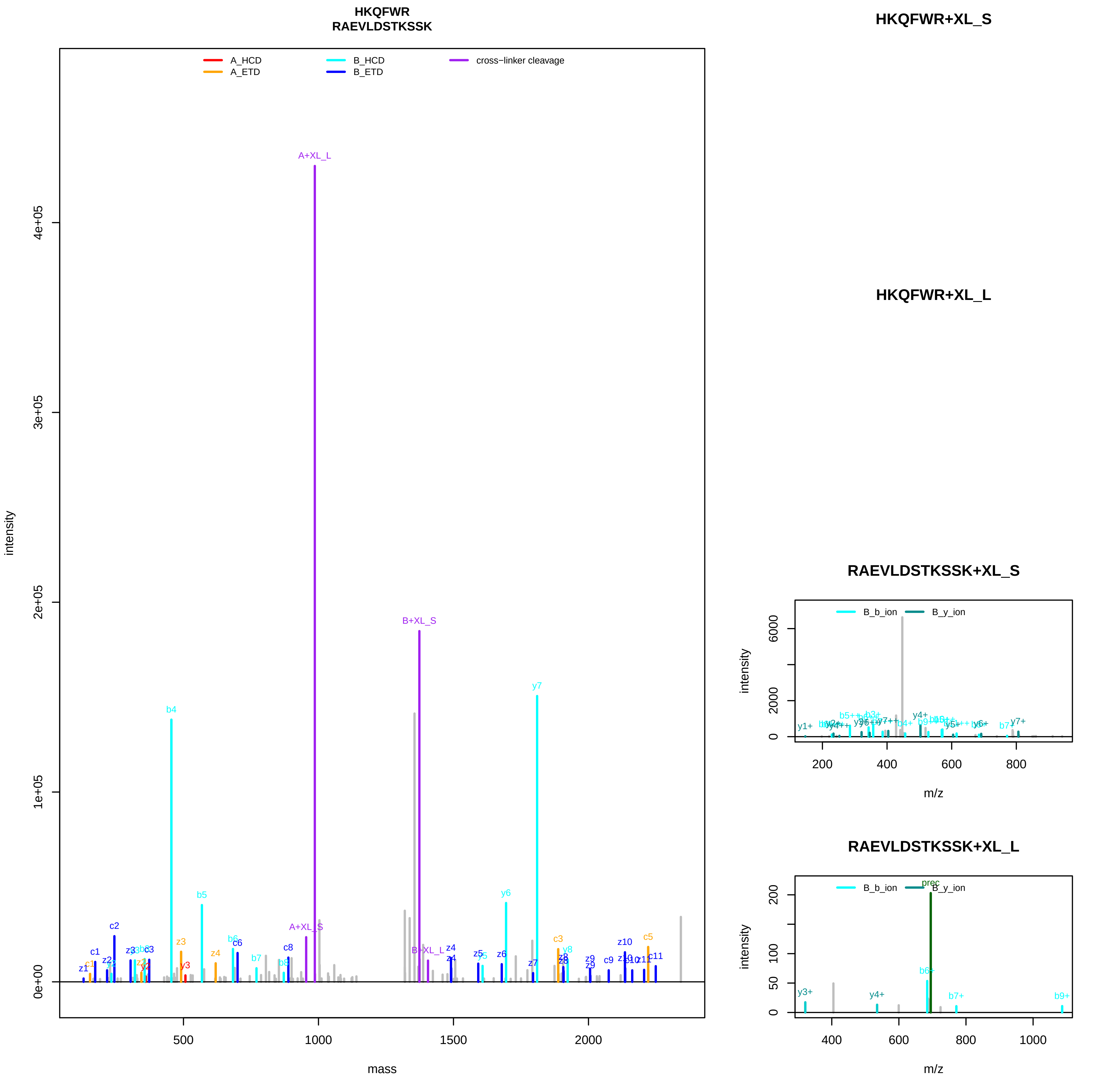
HKQFWR
RAEVLDSTKSSK
A_HCD
A_ETD
B_HCD
B_ETD
cross−linker cleavage
A+XL_L
B+XL_S
A+XL_S
B+XL_L
b4
b5
b6
b7
y6
y7
y8
c2
c3
c5
c6
c8
c9
c11
z3
z4
z5
z6
z7
z9
z10
intensity
mass
0e+00
1e+05
2e+05
3e+05
4e+05
500
1000
1500
2000
HKQFWR+XL_S
HKQFWR+XL_L
RAEVLDSTKSSK+XL_S
B_b_ion
B_y_ion
y1+
y4+
y6+
y7+
b4+
b7+
0
2000
6000
200
400
600
800
m/z
RAEVLDSTKSSK+XL_L
prec
y3+
y4+
b6+
b7+
b9+
0
50
100
200
400
600
800
1000
